# IL-8 and IL-1RA serum levels predicting depression treatment response in 6-week follow-up

**DOI:** 10.1017/neu.2025.10027

**Published:** 2025-07-07

**Authors:** Tuukka Mökkönen, Anssi Solismaa, Mari Hämäläinen, Eeva Moilanen, Olli Kampman

**Affiliations:** 1 Department of Psychiatry, Faculty of Medicine and Health Technology, Tampere University and Tampere University Hospital, Tampere, Finland; 2 Department of Psychiatry, The Pirkanmaa Wellbeing Services County, Tampere, Finland; 3 The Immunopharmacology Research Group, Faculty of Medicine and Health Technology, Tampere University and Tampere University Hospital, Tampere, Finland; 4 Department of Psychiatry, Department of Clinical Sciences (Psychiatry), Faculty of Medicine, University Hospital of Umeå, Umeå University, Umeå, Sweden; 5 Department of Clinical Medicine (Psychiatry), Faculty of Medicine, University of Turku, Turku, Finland; 6 Department of Psychiatry, The Wellbeing Services County of Ostrobothnia, Vaasa, Finland

**Keywords:** Interleukin-8, interleukin-1Ra, depression, follow-up study, inflammation

## Abstract

**Objective:**

This study aims to ascertain the effect of baseline IL-1Ra and IL-8 in the treatment response of patients with major depressive disorder (MDD) and to clarify the relationship between inflammation markers and depression.

**Methods:**

We recruited 242 patients with a Beck Depression Inventory (BDI) score ≥ 17 referred to secondary care in Finland. The patients’ serum IL-1Ra and IL-8 concentrations were measured at baseline. Montgomery-Åsberg Depression Rating Scale (MADRS) tests and Alcohol Use Disorders Identification Tests (AUDIT) were administered at baseline and six weeks. The antidepressant treatments varied: somewere started, others changed or continued their previous medication, and others had their doses adjusted. Patients started behavioral activation therapy. Linear regression was used with a relative MADRS score change during six weeks as the dependent variable and patient age, AUDIT score, BMI, daily number of cigarettes smoked, sex, and serum IL-1Ra and IL-8 concentrations as independent variables.

**Results:**

Higher baseline serum IL-1Ra and IL-8 levels were associated with a smaller relative change in the MADRS-score within the first six weeks of treatment in linear regression analysis (*p* < 0.001 and *p* = 0.007, respectively). In further analysis comparing groups with ≤ 24 and>24 MADRS score only the ≤ 24 MADRS score group showed a similar association.

**Conclusion:**

Higher baseline IL-1Ra and IL-8 concentrations were associated with a lesser relative response to depression treatment, particularly in patients with mild depression. Results on IL-8 concur with earlier findings, whereas the association between higher IL-1Ra serum concentrations reduced treatment response is a novel finding.

## Graphical abstract

**Figure:**
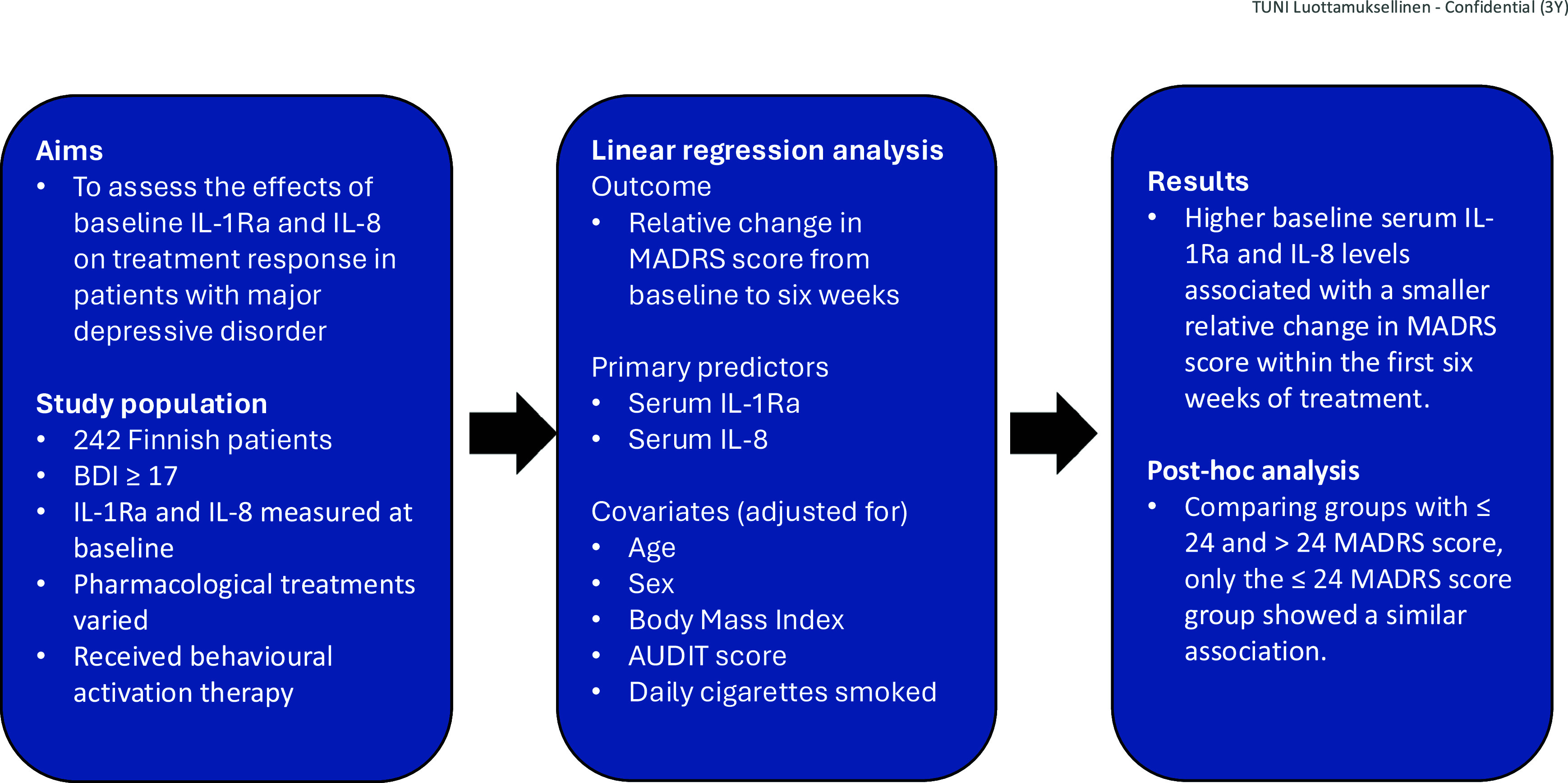

**Significant outcomes**
- Higher baseline IL-1Ra and IL-8 were associated with smaller relative reduction in depression symptoms at the six-week follow-up, particularly in patients with mild depression.
- At the six-month follow-up neither baseline IL-1Ra or IL-8 were associated with symptom reduction.

**Limitations**
- The study population was relatively small (*n*=242).
- The patients were in different stages of treatment at the beginning of the study.

## Introduction

Depression is globally one of the leading causes of disability (World Health Organization, 2023). In addition to better known psychological and social factors contributing to depression such as temperament, personality, and loss of valued relationships, a link between depression and low-grade inflammation has been found (Howren *et al*., 2009; Dowlati *et al*., 2010). Findings suggest that the activation of immunological pathways may play a part in treatment resistant major depression (Raison *et al*., 2006; Miller *et al*., 2009). Interleukin-8 (IL-8) is produced by macrophages and several other cell types in response to pro-inflammatory stimuli. Its main function is the activation and recruitment of neutrophils to the site of inflammation (Akdis *et al*., 2011). Interleukin-1 receptor antagonist (IL-1Ra) is a regulatory cytokine which inhibits interleukin 1 signalling by binding to IL-1 type I receptors. It is synthesised in response to the same stimuli as IL-1 (Akdis *et al*., 2011). In a more recent meta-analysis encompassing 44 studies, Liu JJ et al. explored the association between various cytokines and the response to antidepressant treatment in patients with MDD. The analysis revealed that patients with lower baseline levels of interleukin-8 (IL-8) exhibited a more favourable response to antidepressant therapy (Liu *et al*., 2019). This meta-analysis incorporated findings from nine studies focusing on IL-8, with most indicating a trend; however, the aggregated effect size reached statistical significance. Notably, IL-1Ra was not examined in this meta-analysis. A more recent study reported somewhat contradictory findings: a higher IL-8 serum concentration was associated with a lower severity of depression in female patients with treatment-resistant depression (Kruse *et al*., 2021). A meta-analysis by Howren et al, which included 9 studies, found IL-1Ra to be positively associated with depression (Howren *et al*., 2009). Similarly, a meta-analysis by Köhler et al. reported higher IL-1Ra concentrations in patients with major depressive disorder (MDD) compared to healthy controls (Köhler *et al*., 2017). An association of higher IL-1Ra levels with treatment-resistant depression was also reported by Maes *et al*. (1997). Furthermore, we have earlier observed reductions in depressive symptoms alongside IL-1Ra levels during a six-month follow-up from baseline in a study on depressed patients without uniform pharmacological intervention with behavioural activation as the form of treatment. The results suggest that adiposity may modify the anti-inflammatory response mediated by IL-1Ra in depressed patients. This may be because adipose tissue is a significant source of both IL-1 and IL-1Ra (Archer *et al*., 2022). Another study concluded that there are significant gender-dependent interactions between depression, alcohol use, and mediators of inflammation (Archer *et al*., 2018).

According to current hypotheses, neuropeptide secretion is believed to partially contribute to depression. Corticotrophin releasing hormone is the primary regulator of hormonal and cytokine response to stress. Its elevated secretion has been reliably demonstrated in depressed patients which supports this notion (Raison and Miller, 2003). Meta-analyses have also found evidence about the effects of anti-inflammatory medications like anakinra (IL-1Ra) and non-steroidal anti-inflammatory drugs in depression (Lee and Giuliani, 2019).

The objective of the present study was to investigate whether serum concentrations of IL-1Ra and IL-8 can predict treatment response in MDD. Though past studies have addressed the relationship between IL-1Ra and MDD severity, information about its effect ontreatment response especially in the acute to subacute phase is missing. We also analysed IL-8 and its relationship to treatment response in depression to further elucidate its effects, given the previously mentioned contradictory findings.

## Methods

### Patients and clinical assessments

This study uses data from the Ostrobothnia Depression Study (ODS, 2014). For the study 242 patients were recruited from South Ostrobothnia Psychiatric hospital district, in Finland between 2009 and 2013 by means of referral to secondary care psychiatric services from five outpatient clinics and one inpatient unit. On recruitment patients exhibited depressive symptoms, anxiety, insomnia, self-destructiveness, or alcohol-related problems. Patients were required to have a Beck Depression Inventory score (BDI, Beck and Steer, 1993) of 17 or more for inclusion in the study. Exclusion criteria were a primary diagnosis of psychotic disorders, brain damage or organic brain disease. The psychiatric diagnoses were assessed using the Mini International Neuropsychiatric Interview (MINI, Sheehan *et al*., 1998). At baseline for primary diagnoses 98 (40.5 %) people had depressive disorder, 92 (38.0 %) had recurrent depressive disorder, 9 (3.7 %) had an anxiety disorder, and 18 (7.4 %) had other disorders (Luoto *et al*., 2018). The participants depression severity was measured by using the Montgomery-Åsberg Depression Rating Scale (MADRS, Montgomery and Åsberg, 1979) and information about their alcohol consumption was gathered by means of an Alcohol use disorder identification test (AUDIT, Babor *et al*., 2001). The MADRS- and AUDIT-questionnaires were taken at baseline, 6 weeks and 6 months. Information about the participants smoking habits was gathered at the same intervals as AUDIT score by way of the number of cigarettes per day. Co-morbid substance use was not considered or used as an exclusion criterion in the study due to relatively few patients engaging in illicit substance use. The patients’ body mass index (BMI) was also recorded at baseline, 6 weeks and at six months. Serum inflammation markers for IL-1Ra and IL-8 were measured at baseline and six months later.

Interventions in the study targeted the patient’s alcohol abuse problems and depression depending on how the patient scored in the baseline AUDIT-10-questionnaire. Individuals with a score <11 immediately began psychosocial treatment for depression, whereas individuals who scored ≥11 were first provided motivational interview as well as treatment for their depression. The treatment for depression was realised by using a behavioural activation model by a trained nurse or psychologist in which patients came to a session every 1–2 weeks, with six to eight planned sessions (Paavonen *et al*., 2018; Luoto *et al*., 2021). Upon entering the study, the patients’ antidepressive medications were assessed by a psychiatrist.

During the study, 234 patients were recorded to have antidepressive medication treatments. The medical treatments were not standardised. The medication was assessed at the beginning of the intervention. More detailed information on changes in medication was available for 197 (84.2%) patients: 50 started using antidepressants, 60 had a change in medication or had their dosage increased, 49 continued their earlier dosage, 23 did not or could not use antidepressant medication and 11 started an antipsychotic or a different antidepressant in addition to their pre-existing medication at baseline. The change was unclear for four patients. Fifty patients were recorded to take antipsychotic medication. Of all participants, 82.0 % had either SSRI or SNRI as a primary antidepressant.

After the changes, 93 of the patients were treated with SSRIs, 23 with SNRIs, 22 with other antidepressants, and 68 with a combination of two antidepressants. Thirty-six patients had no antidepressant treatment due to various reasons. All antidepressant doses were converted to their fluoxetine equivalents. The mean fluoxetine equivalent dose for participants was 33.0 mg (SD± 18.3). Further details on the Ostrobothnia depression study can be found on ClinicalTrials.gov under the identifier: NCT02520271 (https://clinicaltrials.gov/ct2/show/NCT02520271?term=NCT02520271&draw=2&rank=1). The study was approved by the local ethics committee and the patients also signed written informed consent.

### Laboratory assays

Venous blood samples were drawn from the subjects at baseline. No standardised time of day was reserved for this but due to regional laboratory operating hours the samples were taken in the morning. IL-1Ra and IL-8 concentrations in serum samples were measured with enzyme-linked immunosorbent assay (ELISA) using reagents from R&D Systems Europe Ltd (Abingdon, UK) and BD Biosciences (Erembodegen, Belgium), respectively. Lowest standards and inter-assay coefficients of variation were 15.6 pg/ml and 7.4% for IL-1Ra and 1.6 pg/ml and 3.7% for IL-8.

### Statistical methods

To account for the differences in patients baseline mood and to measure proportional changes in patients’ depression severity compared to inflammation markers a variable of MADRS score relative change was calculated by subtracting MADRS score at six weeks from the baseline MADRS score, then dividing the result with baseline MADRS score and multiplying the result by 100. This was done to study the percentage change in MADRS score between several timepoints: baseline to six weeks and six months. Furthermore, a dichotomised variable was created: a decrease of 50 % or more in the MADRS score was considered a response (Hirschfeld *et al*., 2002).

In exploratory two-variable comparisons and for detecting multicollinearity between IL-8 and IL-1Ra serum levels at baseline, MADRS relative change, age, sex, AUDIT, BMI, number of daily cigarettes smoked, Pearson correlations and Mann–Whitney U tests were used. Normal Q-Q plots and Shapiro–Wilk test were used to assess distributions of the variables.

To analyse the relationship between serum levels of IL-1Ra, IL-8, and relative changes in MADRS scores, linear regression models were employed. These models used MADRS relative change at six weeks and six months as the dependent variables. Many of the patients were diagnosed with AUD indicating regular alcohol use at the beginning of the study which may influence inflammation markers as found in a study conducted on the same population (Archer *et al*., 2018). For this reason, AUDIT score was included as a coefficient in the regression models. Other covariates included patient age, BMI, number of daily smoked cigarettes, sex and either IL-8 or IL-1Ra as independent variables. Logistic regression was utilised to explore the impact of baseline IL-1Ra and IL-8 on MADRS response (50% reduction) at six weeks and six months, employing a 50% reduction in MADRS scores as the dependent variable. In post hoc analyses, linear regression models were applied to subgroups differentiated by depression severity (baseline MADRS score >24 for moderate to severe, ≤24 for mild), specifically to examine the relative change in MADRS at six weeks, using the median MADRS score as the threshold. Our hypothesis for testing this separately in the mild vs moderate or severe depression was that the group with mild depression is less likely to have multiple psychological and social factors contributing to their depressive symptoms (Nelson *et al*., 2017), so we could possibly observe the inflammatory effect more distinctly in this group with less confounding factors.

The level of significance was set at *p* < 0.05. All statistical analyses were done using SPSS Statistics 28- Statistical analysis software (IBM Corp. Released 2021. IBM SPSS Statistics for Windows, Version 28.0. Armonk, NY: IBM Corp.).

## Results

Table 1 shows the baseline characteristics of the study population. Baseline serum concentrations of IL-1Ra and IL-8 were available from 211(87.2%) patients. MADRS scores were available from 226 (93.4 %) patients at baseline, 182 (75.2 %) at six weeks, and 148 (61.2 %) at six months. Ultimately, 164 patients (67.8%) completed behavioral activation therapy. The median number of therapy sessions patients received was six.

**Table 1.** Baseline characteristics of the study population in the form of mean and standard deviation except in the case of gender which is represented by the percentage of male subjects

AUDIT-scoremean ± sd	10.7 ± 9.9
Cigarettes per day mean ± sd	8.3 ± 9.6
MADRS-scoremean ± sd	23.2 ± 6.7
BMI mean ± sd kg/m^2^	28.2 ± 6.7
IL-1Ramean ± sdpg/ml	831 ± 1316
IL-8 mean ± sdpg/ml	20.5 ± 36.8
Known use of antidepressants during study (%)	80.2
Men (%)	38.8
Agemean ± sd	38.8 ± 12.2

Female patients were slightly younger than male patients (38.1 ± 12.8 years vs. 39.9 ± 11.1 years, *p* = 0,23 Mann–Whitney *U* test). Male patients smoked more cigarettes per day (11.3 ± 11.1 vs. 6.5 ± 8.1 cigarettes, *p* = 0.003, Mann–Whitney *U* test) and had higher baseline AUDIT scores than the female patients (15.1 ± 10.6 vs. 7.9 ± 8.3, *p* < 0.001, Mann–Whitney U test). Of all male patients 68.1 % had an AUDIT score ≥10 whereas the percentage in female patients was 27.0 %. There was no statistically significant difference in baseline IL-8 serum levels between men and women (*p* = 0.534). Baseline IL-1Ra was somewhat higher in women than in men (115.3 and 93.9, *p* = 0.013, respectively)

A significant correlation was found between the number of cigarettes patients smoked per day and baseline AUDIT-score (*r* = 0.405, *p* < 0.001) as well as between baseline MADRS score (*r* = 0.24, *p* < 0.001). Baseline MADRS-score was also found to correlate with the change in MADRS score at six weeks (*r* = 0.43, *p* < 0.001). A correlation between patient age and BMI was also found (*r* = 0.221, *p* < 0.001).

Baseline IL-1Ra was found to correlate significantly with patients’ baseline MADRS-score (*r* = -0.14, *p* = 0.046) as well as the relative change in MADRS score at six weeks (*r* = 0.24, *p* < 0.001).

Baseline IL-8 serum level was found to correlate significantly with BMI (*r* = 0.2, *p* = 0.005). A correlation was also found between IL-8 serum levels and IL-1Ra serum levels at baseline (*r* = 0.49, *p* < 0.001).

Linear regression models explaining the relative change in MADRS score at six weeks are presented in Tables 2 and 3. Similar models explaining the relative MADRS score changeat six months were analysed, but IL-1Ra and IL-8 were not significantly associated with the MADRS changes in these models.

**Table 2.** Linear regression model explaining the percentage change of MADRS score from baseline to six weeks with coefficients: age, AUDIT score at baseline, BMI at baseline, number of cigarettes per day at baseline, gender and IL-1Ra serum level at baseline

	Estimate (*B*)	Std. error	Std. estimate (*β*)	*t*	*p*
Age	0.16	0.25	0.054	0.64	0.53
AUDIT score baseline	0.25	0.4	0.061	0.63	0.53
BMI baseline	0.59	0.5	0.11	1.18	0.24
Number of cigarettes per day	0.16	0.35	0.041	0.45	0.66
Sex	−15.86*	6.79	−0.22	−2.34	**0.021**
IL-1Ra baseline	−0.014**	0.004	−0.34	3.83	**<0.001**

* Negative estimate means that female patients had a higher percentual change towards recovery than male patients.

** Negative estimate means that patients with lower baseline IL-1Ra serum levels had a higher percentual change towards recovery in MADRS-score.

**Table 3.** Linear regression model explaining the percentage change of MADRS score from baseline to six weeks with coefficients: age, AUDIT score at baseline, BMI at baseline, number of cigarettes per day at baseline, sex, and IL-8 serum level at baseline

	Estimate (*B*)	Std. error	Std. estimate (*β*)	*t*	*p*
Age	0.12	0.26	0.039	0.44	0.66
AUDIT score	0.2	0.41	0.048	0.48	0.63
BMI baseline	0.32	0.503	0.058	0.64	0.52
Number of cigarettes per day	0.23	0.36	0.061	0.64	0.53
Sex	−14.75*	7.011	−0.2	−2.1	**0.037**
IL-8 baseline	−0.20**	0.074	−0.24	−2.74	**0.007**

* Negative estimate means that female patients had a higher percentual change towards recovery than male patients.

** Negative estimate means that patients with lower baseline IL-8 serum levels had a higher percentual change towards recovery in MADRS-score.

In binary logistic regression analyses for MADRS response (50% reduction) at six weeks with age, AUDIT- score, BMI, number of cigarettes and gender as independent variables neither baseline IL-1Ra nor IL-8 serum levels showed statistically significant associations.

In post hoc separate group analyses the study population was divided into two groups by depression severity (baseline MADRS points corresponding to mild vs moderate or severe depression) using MADRS relative change as the dependent variable. Negative associations were found in the mild depression (≤ 24 baseline MADRS-score) group with both baseline IL-1Ra (*B* = −0.15, *p* < 0.001) and baseline IL-8 (*B* = −0.208, *p* = 0.016). Neither baseline IL-1Ra nor baseline IL-8 had significant associations with the treatment response in the moderate to severe depression (>24 baseline MADRS-score) group.

## Discussion

The purpose of this study was to find whether IL-8 and IL-1Ra serum levels can predict treatment response in depressed patients. The main finding was that higher baseline serum IL-1Ra and IL-8 levels were associated with a smaller relative depression symptom reduction within the first six weeks of treatment. This can be interpreted that higher IL-8 and IL-1Ra levels could elevate the risk of partial response in depression. Further analysis revealed that the association between IL-8 and IL-1Ra with relative recovery from depressive symptoms was observed only in patients with mild depression, and not in those with moderate to severe depression. Furthermore, when using symptom reduction by half as the dependent variable, neither IL-8 nor IL-1Ra were associated with the outcome at 6 weeks. On a longer six-month timeframe, base IL-8 and IL-1Ra levels were not associated with treatment response.

Our findings regarding serum IL-1Ra levels align with the 1997 study by Maes M et al., which reported higher IL-1Ra serum levels in patients with treatment-resistant depression. Similarly, our study suggests a reduced likelihood of short-term recovery in patients with elevated baseline IL-1Ra. It should be noted, however, that Maes M et al., did not directly compare the percentage change in symptom scores over a follow-up period but rather identified an association between treatment-resistant depression and higher IL-1Ra serum concentrations (Maes *et al*., 1997).

Results regarding serum IL-8 concentration are mostly in agreement with earlier findings. Higher serum IL-8 for instance was found to be associated with higher BMI. An association was found between higher IL-8 serum concentration and weaker response to treatment. The finding is similar in a meta-analysis done by Liu JJ et al., where lower serum IL-8 concentration was associated with greater response to anti-depressant treatment (Liu et al., 2019).This finding is in contrast with a study where changes in IL-8 serum concentration were not found to be significantly associated with response to treatment after 8 weeks of mindfulness based or cognitive behavioural therapy in primary health care (Memon *et al*., 2017).

The primary outcome we focused on was the relative change in MADRS score, chosen because it captures recovery progress regardless of initial severity of depression. Our findings suggest that the impact of IL-1Ra and IL-8 on depression recovery seems more pronounced in patients with mild to moderate symptoms. The IL-1Ra and IL-8 levels seemed to affect treatment response only at the beginning of the follow-up period, as no effect was observed in the six-month analyses. The effect of the IL-1Ra and IL-8 may be so small that it is overshadowed by other factors on a longer timescale. Also, there was a dropout of 34 patients (18.7 %) between the six week and six-month follow-up points, which may affect the results. It is also possible that higher IL-1Ra and IL-8 at baseline slow down depression symptom reduction but have no effect on recuperating from depression on a longer timescale.

This study provides new information on serum IL-1Ra levels’ effect on depression and recovery from depression, where previous research evidence is scarce. There is slightly more literature on the relationship between IL-8 serum levels and depression however more information on the subject is still needed. Accounting patient alcohol use in the analyses is also a notable strength since alcohol use may affect cytokine levels and substance abuse is a common comorbidity in major depression. The analyses on groups separated by baseline MADRS-score give a novel perspective on predicting recuperation from depression since most earlier studies focusing on inflammation markers do not focus on depression severity or recuperation from it. The study also provides relevant longitudinal data on the relationship between inflammatory markers and depression.

Limitations of the study were the relatively small study population and the evaluation of smoking and alcohol consumption through self-reporting questionnaires. Including patients with alcohol use disorder (AUD) in this study can be viewed as both a strength and a limitation. In natural clinical and outpatient settings comorbid alcohol abuse is common among patients with MDD: AUD is the most common disorder to co-occur with MDD and a person with either disorder is twice as likely to also have the other disorder (Boden and Fergusson, 2011). Patient AUD was also accounted for in the regression analyses by including AUDIT-score as one of the variables. The p-values from the exploratory analyses involving two variables were not adjusted using the Bonferroni method and therefore should be interpreted cautiously. Another limitation of the study is its limited clinical relevance; although the impact of the two inflammatory markers on treatment response is statistically significant, the effect size is relatively small. It is important to consider that the study population consisted of patients from secondary healthcare facilities with moderate to severe depression. The effect size observed could differ in a primary healthcare population experiencing a milder form of depression. Furthermore, it should be noted that the patients were not uniform in their phase of depression treatment; some had already commenced antidepressant medication prior to the study baseline, while others had experienced recurrent major depressive episodes. Unfortunately, detailed data on medication changes were unavailable for 15.8% of patients, and pharmacological treatments were not standardised, both of which constitute major study limitations.

Enhancing our understanding of the relationship between inflammatory markers and depression could aid in identifying patients with treatment-resistant depression and in tailoring more effective individualised treatment strategies (Kohler *et al*., 2016). Uher *et al*. (2014) and Jha *et al*., (2017) have studied variability in treatment response with different antidepressants comparing groups with different inflammatory status in the baseline suggesting patients with lower baseline CRP respond better to antidepressant treatment with SSRI:s and those with higher baseline CRP responded better to treatment with nortriptylin or a combination of an SSRI and bupropione (Uher *et al*., 2014; Jha *et al*., 2017). Unfortunately, our sample did not have enough statistical power to compare treatment response within those who started a new antidepressant medication between high and low IL-8 or IL-1Ra levels. Multiple studies have also explored the use of various anti-inflammatory medications, including certain arthritis medications or non-steroidal anti-inflammatory drugs (NSAIDs), for this purpose. A study by Lehrer and Rheinstein for instance found that patients treated with non-steroidal anti-inflammatory drugs report less suicidal ideation (Lehrer and Rheinstein, 2019). A literature review by Maes et al. suggests the use of anakinra (IL-1Ra) medication as a possible treatment for depression (Maes *et al*., 2012). In a small double-blind clinical trial on Sjögrens syndrome patients Anakinra was given as an intervention, a 50 % decrease in fatigue was reported in six of the 12 patients in the intervention group compared to the placebo group where only one of the 13 patients showed the same effect (Norheim *et al*., 2012). At first glance the results of the Norheim et al., study seem to conflict with our study’s result. However endogenous IL-1 and IL-1Ra are released in response to the same stimulus so it is possible the IL-1 that was not measured in this study is attributing to these findings. The function of IL-1Ra is to modulate the effects of IL-1. Since no exogenous IL-1Ra was administered to patients in this study the measured IL-1Ra is released in proportion to IL-1 unlike in the Norheim et al., study where IL-1Ra was added exogenously increasing the modulation of IL-1’s effects. Current treatment guidelines do not support the use of anakinra or NSAIDs in treatment of depression as the evidence is scarce. Further data are required to determine whether anti-inflammatory medications could be effective in treating a specific subgroup of patients with depression.

## Conclusions

We found that higher baseline IL-1Ra and IL-8 serum concentration predicted a smaller percentage change towards remission in MADRS-score in patients with mild depression within a six-week time frame. However, this effect was relatively small and not observed in analysis where treatment response, defined as *a* ≥50 % decrease in MADRS-score, was used as the outcome.
